# TEAD4 functions as a prognostic biomarker and triggers EMT via PI3K/AKT pathway in bladder cancer

**DOI:** 10.1186/s13046-022-02377-3

**Published:** 2022-05-17

**Authors:** Ming Chi, Jiao Liu, Chenxue Mei, Yaxing Shi, Nanqi Liu, Xuefeng Jiang, Chang Liu, Nan Xue, Hong Hong, Jisheng Xie, Xun Sun, Bo Yin, Xin Meng, Biao Wang

**Affiliations:** 1 Department of Biochemistry and Molecular Biology, School of Life Sciences, China Medical University, Shenyang, 110122 China; 2 Department of Urology, the First Affiliated Hospital of China Medical University, Shenyang, 110001 Liaoning China; 3 Department of Gastroenterology Medicine, Shengjing Hospital of China Medical University, Shenyang, 110004 China; 4 Department of Urology, ShengJing Hospital of China Medical University, Shenyang, China; 5 Institute of Health Science, China Medical University, Shenyang, 110122 China; 6 Department of Immunology, College of Basic Medical Sciences of China Medical University, Shenyang, China; 7 Department of Radiation Oncology, the First Affiliated Hospital of China Medical University, Shenyang, China; 8 Department of Orthodontics, School and Hospital of Stomatology of China Medical University, Liaoning Provincial Key Laboratory of Oral Disease, Shenyang, China; 9 Department of Geriatrics, The First Affiliated Hospital of China Medical University, Shenyang, China; 10 Department of Histology and Embryology, Youjiang Medical College for Nationalities, Baise City, China

**Keywords:** TEAD4, EMT, Bladder cancer, PI3K, AKT

## Abstract

**Background:**

The distant metastasis is the primary cause of cancer morbidity and mortality for bladder cancer (BLCA) paitents. All the recommended therapy for it largely depends on how far the cancer has invaded. It has been confirmed that epithelial to mesenchymal transition (EMT) is the leading reason for the BLCA metastasis which makes BLCA difficult to cure. The aim of the present study is to identify the BLCA-related genes that can be used as the new prognostic biomarker and treatment target, and to investigate the functional mechanisms of TEAD4 in EMT dysregulation.

**Methods:**

The "limma" R package was used to identify the differentially expressed genes (DEGs) between the normal and the tumor samples from TCGA BLCA and GTEx databases. Kaplan–Meier and UniCox analysis were used to filter DEGs with prognostic value in BLCA. Step muti-Cox analysis was used to construct a prognostic risk score model based on clinical phenotype characters. Gene set enrichment analysis (GSEA) was performed to explore the possible molecular mechanisms affecting the prognosis in BLCA. Unsupervised hierarchical clustering analysis was performed to evaluate the effects of EMT process on the prognosis. Single-sample GSEA (ssGSEA) was used to calculate the correlation betweeen the expression of DEGs and EMT enrichment scores. TEAD4 expression and its association with pathological grading and survival were appraised in samples from TCGA dataset and BLCA tissue microarray. Colony formation assays and CCK8 assays were performed to study the changes in BLCA cell proliferation when the TEAD4 levels was down- or up-regulated in BLCA cells. Transwell and wound healing assays were utilized to analyze the impact of TEAD4 on the invasion and metastasis of the BLCA cells. Western Blot was carried out to detect the changes of EMT-related markers and the active molecules involved in PI3K/AKT signaling in BLCA cells. Kyoto Encyclopedia of Genes and Genomes (KEGG) enrichment analysis was conducted on the genes related to TEAD4 expression. 740Y-P (activator of PI3K/AKT pathway) and LY294002 (inhibitor of PI3K/AKT pathway) were applied to evaluate the contribution of PI3K/AKT signaling pathway in the EMT of BLCA cells. To examine the in vivo effect of TEAD4 on tumor metastasis, we designed a metastatic nude-mouse model by tail vein injection of TEAD4-knockdown BLCA cells. And PET/CT imaging was used to assess the extent of lung metastases.

**Results:**

A total of 1592 DEGs were recognized, among which 4 DEGs have been identified as independent prognostic factors for BLCA, such as FASN, IGFL2, PLOD1 and TEAD4. TCGA BLCA samples were divided into significantly different low- and high-risk groups according to the median risk score; GSEA analysis showed that HALLMARK EMT pathway was the top enriched gene signature when compared high-risk and low-risk groups, which was also verified by unsupervised cluster analysis. EMT signature-derived ssGSEA scores demonstrated that TEAD4 had the most positive correlation with EMT process. In addition, TEAD4 expression was upregulated in TCGA BLCA samples and correlated with pT stage, tumor stage and tumor grade. Functional studies showed that TEAD4 knockdown via lentiviral TEAD4 shRNA inhibited cell migration and invasion in vitro and in vivo, with the reduced expression of EMT related markers in BLCA cell lines; the migration and invasion of TEAD4 knockdown cells could be restored by ectopic expression of TEAD4. Meanwhile, KEGG enrichment analysis of genes related to TEAD4 expression showed that enrichment was significantly related to PI3K/AKT pathway. The pathway inhibitor LY294002 blocked the TEAD4-induced enhancement of migration and invasion as well as the expression EMT-related markers, whereas the agonist 740Y-P rescued the decreased migration, invasion and EMT induced by TEAD4 knockdown.

**Conclusions:**

TEAD4 is closely correlated with poor prognosis in BLCA and mediates its metastasis through regulating EMT via PI3K/AKT pathway, proving that TEAD4 is not only an effective biomarker for predicting the prognosis but also a great potential target for treatment of metastatic BLCA.

**Supplementary Information:**

The online version contains supplementary material available at 10.1186/s13046-022-02377-3.

## Introduction

Bladder cancer (BLCA) is the malignant tumor of the urinary system with high morbidity and mortality, which is reported as the 10th most common cancer with more than 430,000 new cases every year in the word [1, 2]. BLCA is characterized as a heterogeneous disease consisting of two major subtypes, non-muscle-invasive bladder cancer (NMIBC) and muscle-invasive bladder cancer (MIBC). About 70–80% of BLCA patients are initially diagnosed with NMIBC [3]. Although the prognosis of NMIBC patients has been greatly improved, more than 60% of NMIBC patients would recur and more than 20% would deteriorate to MIBC with high mortality and metastasis rate [1, 4]. The number of therapeutic options for the treatment of invasive and metastatic BLCA is quite limited, which has become a huge clinical challenge. Thus, it is urgent and crucial to identify novel molecular targets for controlling invasion and distant metastasis of BLCA.

Epithelial—mesenchymal transition (EMT) is the process of lineage transition between epithelium and mesenchyme, by which the polarized epithelial cells lose their adhesive properties and obtain mesenchymal cell phenotypes [5]. This transition is characterized by the increase of mesenchymal markers such as snail, N-cadherin and vimentin; and the downregulation of the epithelial markers such as E-cadherin, Zonula occludens-1 (ZO-1) and occludin [6, 7]. EMT process is widely involved in a series of biological processes and is thought to be closely related to the invasion and metastasis progression of tumors [7]. In most of human carcinomas, the cancer cells undergo EMT by which the cells lose their cell polarity and cell–cell adhesion and gain migratory and invasive abilities [8, 9], enabling them to proliferate and metastase ultimately following extravasation [10]. Previous studies have proved that EMT contributed to bladder cancer progression [11]. Recently, a cohort analysis showed that EMT was a key factor in the subtype transition from NMIBC to MIBC in BLCA using the data from Gene Expression Omnibus (GEO) and The Cancer Genome Atlas (TCGA) data sets [12]. However, the regulatory network for the activation of EMT in BLCA remains elusive.

TEA domain (TEAD) transcription factors play important roles in cell proliferation, tissue regeneration, and stem cell maintenance [13, 14]. TEAD4 is a member of the TEADs family, which functions by interacting with transcriptional co-activators [15]. In recent years, TEAD4 has become a new prognostic and predictive molecular marker for various types of cancer as roles of TEAD4 in tumor development are being gradually discovered. A study in head and neck squamous cell carcinoma (HNSCC) showed that TEAD4 expression promoted invasion, migration and EMT of HSECC cells and was significantly associated with poor prognosis in HNSCC patients [16]. Another study in lung adenocarcinoma found that TEAD4 hardly affected the proliferation, cell cycle and apoptosis of lung adenocarcinoma cells, but it could significantly enhance the invasion and migration via the EMT pathway in the cancer cells [17]. In BLCA, only one bioinformatic analysis of the gene expression profiles from GEO and TCGA showed that TEAD4 could be a prognostic biomarker promoting the cell migration and invasion via EMT [18]. But the role of TEAD4 and the detail mechanism by which TEAD4 promotes EMT in BLCA cells have not been reported yet.

It's well known that PI3K/AKT signaling pathway plays an important role in regulating fundamental cellular functions and metabolism. In cancers, PI3K/AKT pathway maintains the biological characteristics of malignant cells [19, 20] and induces EMT directly or through cooperation with other signaling pathways [21–24], promoting the invasion and metastasis of tumor cells. Recently, it was documented that PI3K/AKT pathway was invovled in the enhanced metastasis and EMT induced by lncRNA ADAMTS9-AS1 [25] and C19orf10 [26] in BLCA cells. In a similarily way, many molecues sponsored migration, invasion and EMT of BLCA cells in-vitro and in vivo through PI3K/AKT pathway, such as CERCAM [27]  and GAS6 [28].  On the contrary, some other molecules such as TSPAN7 [29] and ID2 [30] inhibited the cell migration and invasion via PI3K/AKT. All these studies have proved the particular importance of PI3K/AKT pathway in mediating cell metastasis of BLCA.

**Fig. 1:**
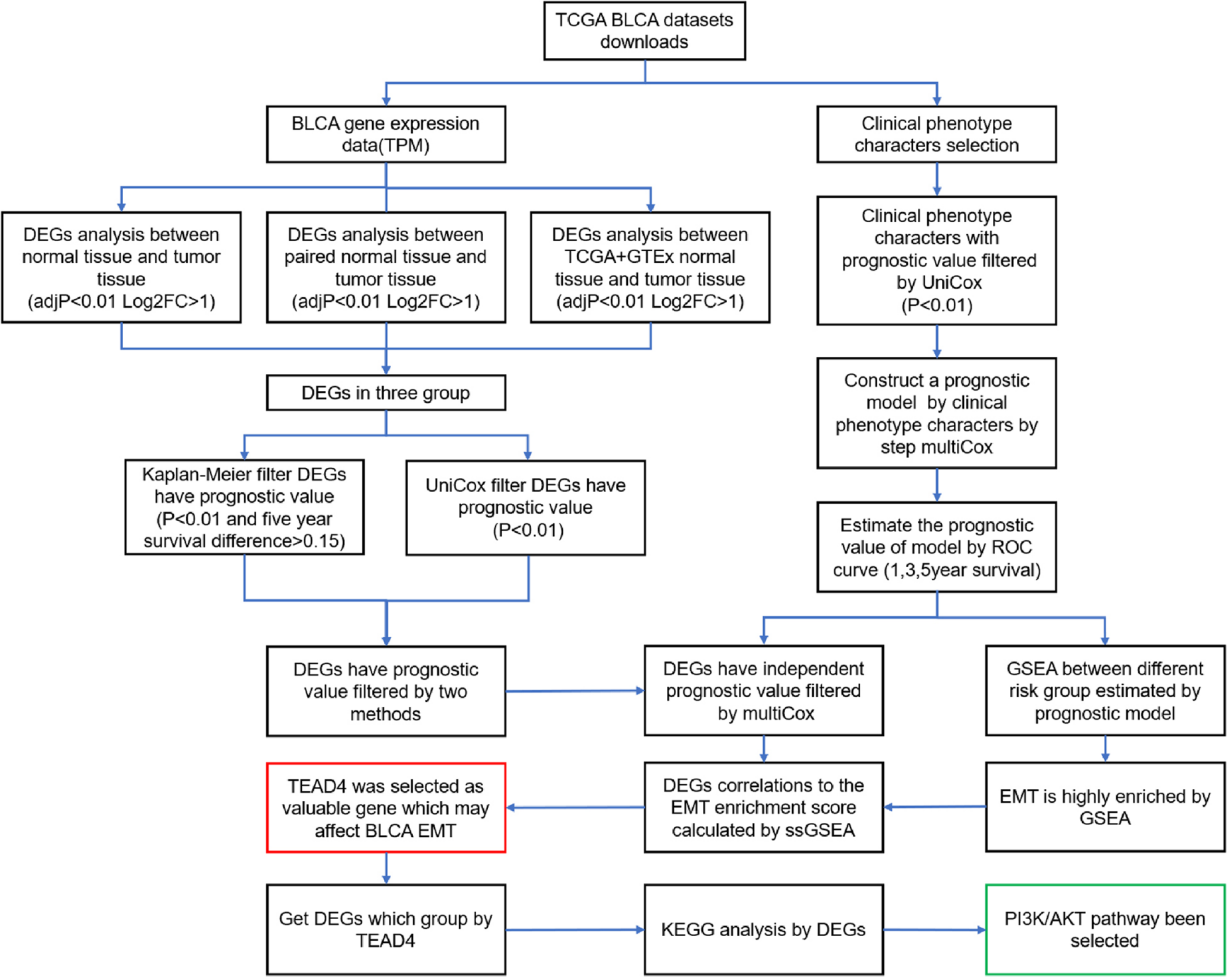
Analysis flowchart

In the present study, we systematically analyzed the relationship between the differential expressed genes (DEGs) and the clinicopathological characteristics of 433 BLCA patients from TCGA and GTEx database (Fig. 1). And we identified four independent prognostic factors for prognosis of BLCA patients. Then, a prognostic risk score model was built via multivariable Cox regression analysis based on clinicopathological features. Using the median risk score as the cut-off point, TCGA BLCA samples were divided into high-risk group and low-risk group. We found only EMT-related gene set was significantly high-enriched in the high-risk group. Then, the cluster analysis was performed on the EMT-related gene profiles. We found that the patients in high EMT expression group had a significantly shorter survival time than those in low EMT expression group, proving that EMT was a key biological process for poor prognosis in BLCA. By ssGSEA, we found that TEAD4 was the most strongly associated with EMT among the four independent prognostic factors. Enforced expression of TEAD4 stimulated the metastatic potential of BLCA cells via induction of the EMT process. In addition, we revealed that TEAD4 activated PI3K/AKT pathway, thereby contributing to EMT in BLCA cells.

Taken together, our data indicated that TEAD4 promotes BLCA metastasis through PI3K/AKT pathway mediated activation of EMT and can be used as a promising prognostic factor and therapeutic target in BLCA.

## Materials and methods

### Data source

The genomic data and the corresponding clinical data of BLCA were downloaded from the UCSC Xena database (http://xena.ucsc.edu/). Different batches of sample data from the same patient were removed and 424 samples data were finally collected (from 405 cases of tumor tissue and 19 cases of adjacent tissue). And the genomic profiles of 9 healthy bladder tissues were downloaded from the Genotype-Tissue Expression (GTEx) database (https://www.gtexportal.org/home/index.html). Data analysis were carried out with the R Programming Language, version 4.0.5. tidyr, dplyr, rtracklayer R packages were used for data cleaning and ID transformation.

### DEGs screening

DEGs were screened using the Limma R package. Based on Bayesian calculation of T-values, F-values and log-odds, the eligible DEGs were selected using the criteria of |log2(FC)|> 1 and adj *P* Value < 0.01. All the data were visualized by plotting volcano plots using the ggplot2 package in R.

### Independent prognostic analysis of DEGs

Kaplan–Meier survival and Univariate Cox analyses were used to screen prognostic DEGs with *P* Value < 0.01 and five-year overall survival (OS) difference >|0.15|. The genes that are both required for the screening methods and whose increased expression is consistent with prognostic risk are selected. A prognostic risk model based on the gene expression was constructed and used as well as multivariate Cox regression analysis to screen DEGs with independent prognostic value, and *P*-value < 0.01 is set as the cutoff criterion.

### Gene set enrichment analysis (GSEA)

The prognostic model based on pT Stage, pN Stage and Age was constructed using Step Multivariate Cox. Then, the genomic data of 335 patients in the TCGA database were divided into high-risk group and low-risk group depending on the risk score. The hallmark (h.all.v7.2.symbols.gmt) gene set was used for the enrichment analysis in GSEA (Gene Set Enrichment Analysis) v 4.1.0 software (https://www.gsea-msigdb.org/gsea/index.jsp). The high-risk group versus low-risk group was used as the phenotypic label and the number of permutations was set to 1000. All other options were set as default.

### Non-negative matrix factorization (NMF) clustering

The HALLMARK_EPITHELIAL_MESENCHYMAL_TRANSITION gene set was used for NMF clustering. “NMF” R package was used to perform unsupervised NMF clustering with 1,00 repeat samples and a maximum grouping of 5 on the metadata set. The NMF rank survey and consensus heatmap were used to evaluate the optimal k value, and the TCGA samples were divided into 2 clusters according to the EMT correlations degree. Kaplan‐Meier survival analysis was used to assess the survival differences between different clusters.

### Single sample GSEA (ssGSEA)

The gene list of HALLMARK_EPITHELIAL_MESENCHYMAL_TRANSITION from the GSEA database was downloaded and used for the ssGSEA analysis with GSVA and GSEABase R packages. The results were plotted by ggplot2 package in R.

### Kyoto Encyclopedia of Genes and Genomes (KEGG) enrichment analysis

Four hundred twenty-four BLCA patients were classified into high-/low- TEAD4 subgroup based on the median TEAD4 expression level. The limma package was used to get DEGs between the two groups. The current filter criteria of *P* < 0.01 and |log_2_FC|> 1 was set. KEGG pathway enrichment analysis was performed with DAVID Bioinformatic Resources 6.8 server. The results were visualized using ggplot2 package in R.

### Correlation analysis among the TEAD4 expression and clinicopathological characteristics

Package ggpubr was loaded to perform the correlation analysis of TEAD4 mRNA expression with clinicopathological characteristics. Boxplots were used to show the relationship between the TEAD4 expression and the corresponding clinicopathological characteristics, including pT stage, tumor stage and tumor grade.

### Preparation of lentiviral shRNA targeting TEAD4

Lentiviral particles were produced in 293 T cells using polyethylenimine (PEI) (Sigma-Aldrich). In brief, 293 T cells were co-transfected with 2.5 μg of pMD2.G (envelope expressing plasmid), 7.5 μg of psPAX2 (lentiviral packaging plasmid) and 10 μg of RNAi plasmid (Genechem, Shanghai, China). 293 T supernatant was harvested after 36 or 48 h of transfection and filtered by 0.45 μm membrane (Millipore, USA). The RNAi sequence was provided in Supplementary Table 1.

### Tissue microarray (TMA) and immunohistochemistry (IHC)

The bladder cancer tissue microarray (TMA) consisted of 63 BLCA specimens (Shanghai Outdo Biotech Co., Ltd.) was used for the IHC analysis. IHC staining was performed using antibody to TEAD4 (1:100, Bioss, bs-9603R). The TMA slides were scored manually through visual assessment with three independent researchers blinded to clinicopathologic information. Discrepancies were resolved by discussion. The expression of TEAD4 was assessed according to the immunoreactive score (IRS), which was obtained as a product of multiplication between positive cells proportion score (0—4: 0: 0%, 1: 1∼25%, 2: 26∼50%, 3: 51∼75%, 4: 76∼100%) and staining intensity score (0—3: 0: negative, 1: weak, 2: moderate and 3: strong).

### Cell culture and transfection

SV-HUC-1, TCCSUP, 5637, BIU-87, T24, 293 T cells were purchased from the Chinese Academy of Science (Shanghai, China). Cells were cultured in RPMI-1640, DMEM or Ham’s F-12 medium (Procell Co., Ltd, China) and supplemented with 10% FBS (Biological Industries, Israel) at 37 °C in a 5% CO_2_ humidified atmosphere. The TEAD4 was overexpressed in the BLCA cells by transfection with the plasmid (Genechem, Shanghai, China) in Polyplus Invivo-jetPEI (Polyplus, French) reagent according to the instructions recommended. To construct of TEAD4 stable knockdown cells lines, 5637 and T24 cells in logarithmic growth phase were transfected by lentivirus, following by culturing in 1640 medium with 10% FBS in a 6-well dish. Puromycin (2 μg/μl) was added for selection when the cell density reached 90%, and the stable colnoies will be amplified after 10–14 days. The overexpression/knockdown efficiency of TEAD4 was evaluated by qPCR and western blot.

### Colony formation assay

The 500 transfected cells were seeded in 6-well plates in culture medium containing 10% FBS for about 2 weeks. The cell colonies were fixed with 4% paraformaldehyde for 30 min and then stained with 0.1% crystal violet the other 30 min. Finally, a high-definition digital camera was used to take pictures and then analyzed by ImageJ software.

### Cell Counting Kit-8 assay

The cells were seeded into 96-well plates at a density of 2000 cells/well and maintained for 24 h at 37° C in 5% CO_2_. At the specified time, 10 μl CCK8 solution was added to each well. Tree hours later, the absorbance was detected by a multi-scan spectrophotometer at 450 nm.

### Migration and invasion assays

Trans-well membrane (Corning 3422, 8 μm pore size) with or without Matrigel was used to evaluate the invasion or migration ability of BLCA cells. Briefly, 2–4 × 10^4^ cells were inoculated into the upper chamber containing 200 μl FBS-deficient medium. Meanwhile, medium (600 μL) containing 10% FBS was added to the lower chamber. After incubating for 24 h, 36 h or 48 h at 37 °C, the chambers were washed with PBS and fixed by 4% paraformaldehyde for around 30 min. The cells on the upper side of the membrane were scraped with a cotton swab and stained with crystal violet for about 30 min at room temperature. The membranes were washed in PBS and photographed after dried out.

### Wound healing assay

For wound-healing assay, the cells were seeded in a 6-well plate. The cell layer is scratched with a sterile plastic suction pipette. Subsequently, the cells were cultured in FBS-deficient medium, and the images were acquired by electron microscope at 0 h, 12 h, 24 h and 48 h, respectively. The migration ability of cells is evaluated by measuring the changes in the size of the injured area.

### Total RNA isolation and quantitative RT-PCR

TRIZOL reagent (TaKaRa, Japan) was used for extracting total RNA from cells. According to the kit instructions, the cDNA was synthesized using the quantified.

RNA as a temple with reverse transcription kits (Takara, Japan). Real-time quantitative PCR was performed by Roche LightCycler 480 II system (Roche, Basel, Switzerland). The fold changes were calculated according to the formula 2^−ΔΔCt^ method. The primer sequences used were provided in Supplementary Table 2.

### Western blot

Cells were lysed in RIPA lysis buffer (Beyotime Biotechnology, China) containing protease inhibitor cocktail. BCA protein quantification kit (Vazame, China) was used to detect the protein concentration. Equal amounts of protein were separated by SDS-PAGE and then transferred to PVDF membrane. After blocking with 5% BSA for 1 h, the membranes were incubated overnight at 4 °C with the indicated primary antibodies, followed by incubation with HRP-labeled secondary antibody for 1 h at room temperature. Primary antibodies were purchased from Cell Signaling Technology (E-cadherin, 1:1000, #3195; N-Cadherin, 1:1000, #13,116; Snail, 1:1000, #3879; MMP-2, 1:1000, #40,994; MMP-9, 1:1000, #13,667; Phospho-Akt, 1:1000, #4060; Akt, 1:1000, #4691; PI3K, 1:1000, #3011), Bioss (Phospho-PI3KCA, 1:1000, bs-5570R), Proteintech (TEAD4, 1:2000, 12,418–1-AP; Vimentin, 1:1000, 10,366–1-AP), and ImmunoWay (GAPDH, 1:10,000, YM3209). Secondary antibodies were purchased from Elabscience (Goat Anti-Rabbit IgG (H + L), 1:10,000, E-AB-1003; Goat Anti-Mouse IgG(H + L), 1:10,000, E-AB-1001).

### In vivo studies

This study has been approved by the Animal Care and Use Committee of China Medical University (NO. CMU2020398). Four-to-Five-week-old female nude mice (BALB/c-nu) were purchased from Beijing SIPEIFU Biotechnology Co., Ltd., and were raised in the Specific Pathogen Free (SPF) feeding condition of the Department of Laboratory Animal Science of China Medical University. The LV-NC-transfected T24 cells and the LV-shTEAD4-transfected T24 cells (1 × 10^6^ resuspended in 200 μl PBS) were injected into the tail vein of nude mice (*n* = 6). Eight weeks later, the mice were anesthetized with phenobarbital sodium and the contrast media (18F-FDG) was injected. The mice were scanned and imaged with a small animal PET scanner (MadicLAB). Finally, all nude mice were sacrificed, and the lungs were dissected out and photographed.

### Statistic

Values are expressed as mean ± SD. We used ggplot2 R Package and GraphPad Prism 8 to create statistic diagrams for results. Generally, *P* Values < 0.05 was considered as a statistical significance. All experiments in this study were repeated three times.

## Results

### Identification and screening of DEGs

**Fig. 2:**
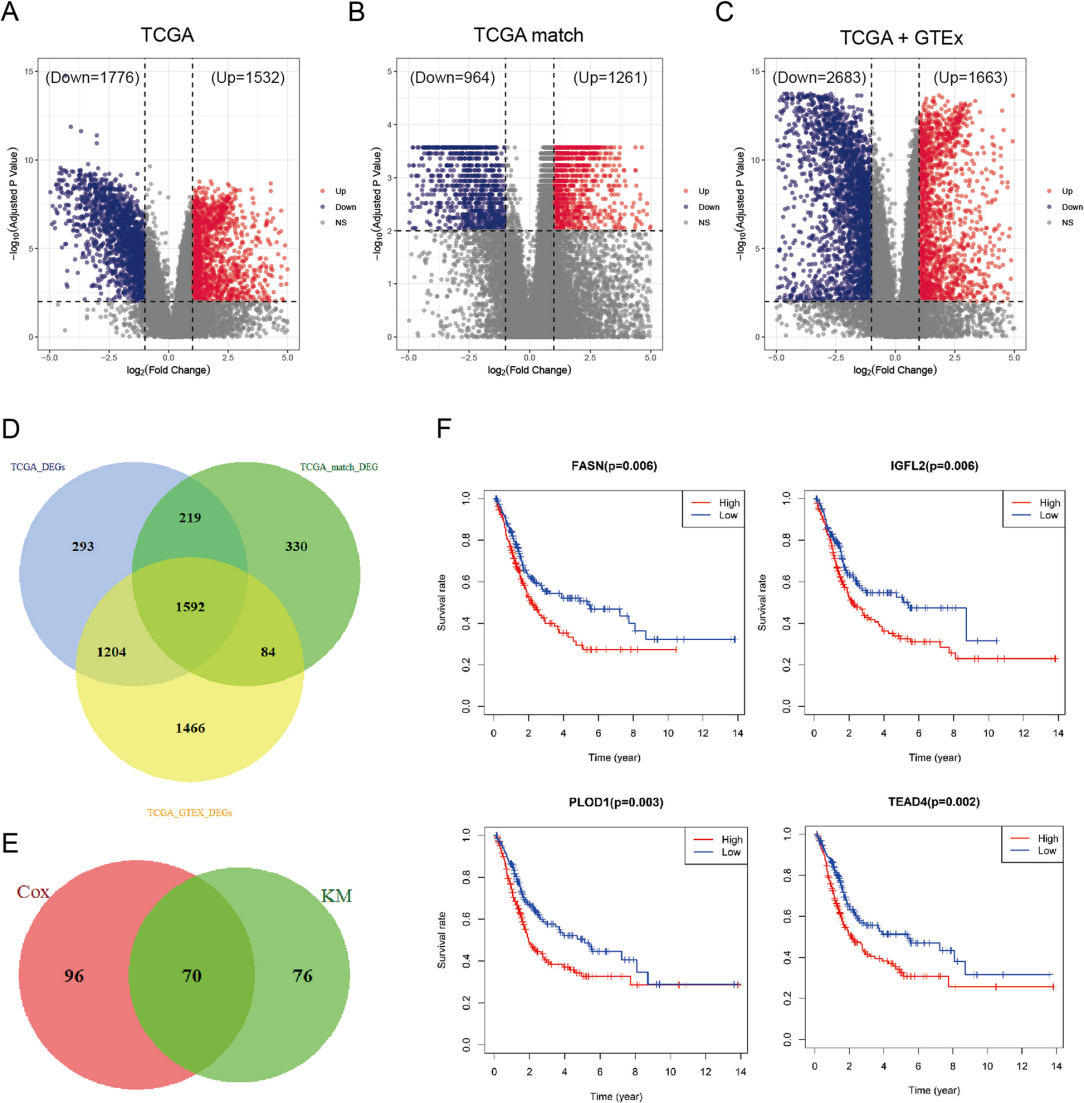
Screening of DEGs in BLCA (**A**) The volcano plot shows the upregulated and downregulated DEGs in the BLCA tissues compared to normal bladder tissues from TCGA database. (**B**) The volcano plot shows the upregulated and downregulated DEGs in 19 pairs of BLCA samples and their matched adjacent normal tissue selected from TCGA database. (**C**) The volcano plot shows the upregulated and downregulated DEGs in the combined expression data from GTEx and TCGA database. (**D**) Venn diagram of the DEGs screened by three analytic methods, revealing 1592 intersected genes. (**E**) Venn diagram of the prognosis—associated genes selected by KM and Cox analyses, revealing 70 intersected genes. (**F**) Survival curves of patients from TCGA-BLCA database with high or low (FASN, IGFL2, PLOD1 or TEAD4) expression levels

To better understand the molecular basis associated with BLCA, first we analyzed the gene expression profiles in 424 BLCA patients from TCGA database. The results showed that 1532 genes were upregulated and 1776 genes were downregulated in BLCA tissues compared to normal bladder tissues (Fig. 2A, adj *P* < 0.01, |log_2_ FC|≥ 1). Subsequently, we analyzed the expression profiles of mRNAs on 19 pairs of BLCA samples and their matched adjacent normal tissue selected from TCGA database. The results show that 1261 genes were upregulated and 964 genes were downregulated in BLCA tissues compared to their paired normal tissues (Fig. 2B, adj *P* < 0.01, |log_2_ FC|≥ 1). Finally, we systematically analyzed the combined expression data from GTEx and TCGA database and found that there were 1663 genes upregulated and 2683 genes downregulated in BLCA tissues compared to normal bladder tissues (Fig. 2C, adj *P* < 0.01, |log_2_ FC|≥ 1). Finally, we defined a core set of 1592 DEGs that were identified by the three analytic methods (Fig. 2D).

To further clarify the relationship between the expression of the identified DEGs and the prognosis of BLCA patients, both Kaplan–Meier and univariate Cox regression analyses were performed using the “survival” R package for screening potential prognostic genes. Only genes that satisfied the two criteria were considered true prognosis—associated genes. The results indicated that only 70 genes were significantly associated with the prognosis of BLCA patients (Fig. 2E). Among them, the genes that their expression abundance was negatively correlated with the prognosis were included for the following analysis. Only 4 genes of FASN, IGFL2, PLOD1 and TEAD4 were identified (Fig. 2F) as prognostic factors for our subsequent study.

### EMT is a key factor contributing to poor prognosis in BLCA

In order to find the clinical factors leading to the poor prognosis of BLCA, the expression profiling dataset and the corresponding clinical information from TCGA were used here. First, clinical features with prognostic value were filtered by univariate Cox. The results suggested that Age, pM Stage, pN Stage, pT Stage and Tumor Stage had prognostic value in BLCA (*P* < 0.01).

**Fig. 3:**
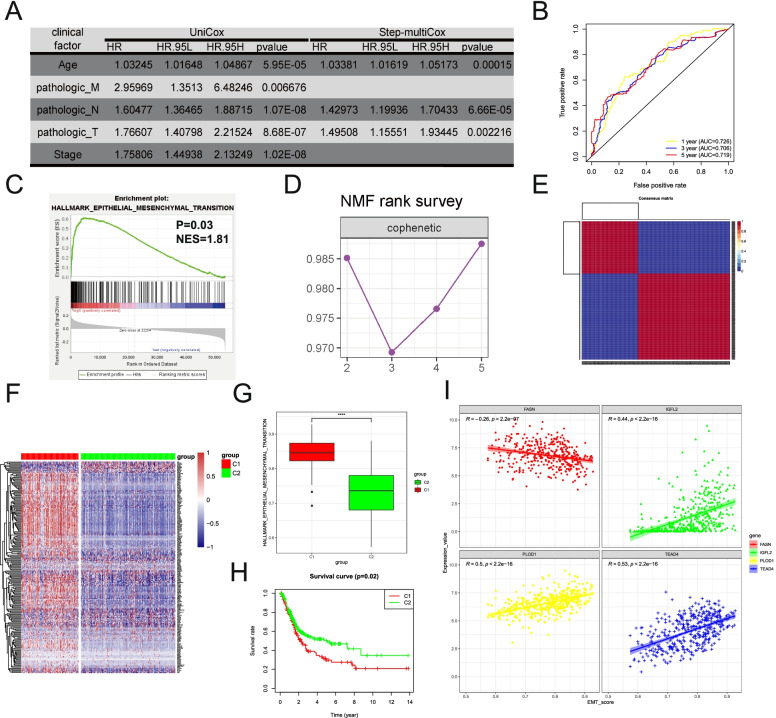
EMT is a key factor contributing to poor prognosis in BLCA (**A**) Univariate and multivariate Cox analysis results of Clinical phenotype characters in TCGA-BLCA database (**B**) Time‐dependent ROC curve of BLCA prognostic model. The AUC was assessed at 1, 3 and 5 years. (**C**) GSEA results of the high-risk group versus low-risk group in prognostic model (*P* = 0.03, NES = 1.81). (**D**) NMF rank survey of unsupervised clustering results. (**E**) Consensus matrix heatmap of two clusters yielded by the unsupervised clustering. (**F**) Enrichment of EMT related gene sets in the two clusters yielded by the unsupervised clustering. (**G**) Boxplots showing the enrichment levels of EMT-related genes in Cluster1 and Cluster2 groups (**H**) Survival curves of patients from Cluster1 and Cluster2 (*P* = 0.02). (**I**) Scatter plot of the correlation between prognostic factors (FASN, IGFL2, PLOD1 and TEAD4) expression and EMT in BLCA

To determine whether the prognostic value of the 4 genes (FASN, IGFL2, PLOD1 and TEAD4) was independent of these clinical factors, multi-Cox regression were performed. The results suggested that all four genes can be used as independent prognostic factors for BLCA (Supplementary Table 3). In the meantime, Age, pN Stage and pT Stage were also proved to be as the independent prognostic factors (Fig. 3A). Then, we established a prognostic risk score model based on these three clinical factors; using the median risk score as the cut-off point, TCGA BLCA samples could be divided into significantly different low- and high-risk groups. Time-dependent ROC analysis showed that the prognostic accuracy of this model was 0.726 at 1 year, 0.706 at 3 years and 0.719 at 5 years (Fig. 3B).

Based on this model, GSEA analysis showed that HALLMARK EMT pathway was the top enriched gene signature (*P* = 0.03, NES = 1.81) in the high-risk group (Fig. 3C and Supplementary Fig. 1). Subsequently, unsupervised cluster analysis was conducted using the HALLMARK_EPITHELIAL_MESENCHYMAL_TRANSITION gene set in the GSEA on the BLCA data from TCGA (non-negative matrix analysis, NMF, repeated calculations 100 times). By comparing the different number of clusters, 2 clusters were selected as an acceptable criterion (Fig. 3D and E, supplementary Figs. 2 and 3). The EMT-related genes expression was highly enriched in the cluster1 compared to those in the cluster2 (Fig. 3F and G). The patients in cluster 1 had obviously shorter OS compared to patients in cluster 2 (Fig. 3H). The above results suggested that EMT was a key factor leading to the poor prognosis of BLCA patients.

Finally, EMT signature-derived ssGSEA scores displayed a strong correlation with the expressions of FASN, IGFL2, PLOD1 and TEAD4. Among the four genes, TEAD4 had the strongest positive correlation with EMT in BLCA (*R* = 0.53, *P* < 0.01) (Fig. 3I). Therefore, TEAD4 was selected as the target-of-interest in our following validation experiments.

### TEAD4 is highly upregulated in BLCA and correlates with clinical outcomes

**Fig. 4:**
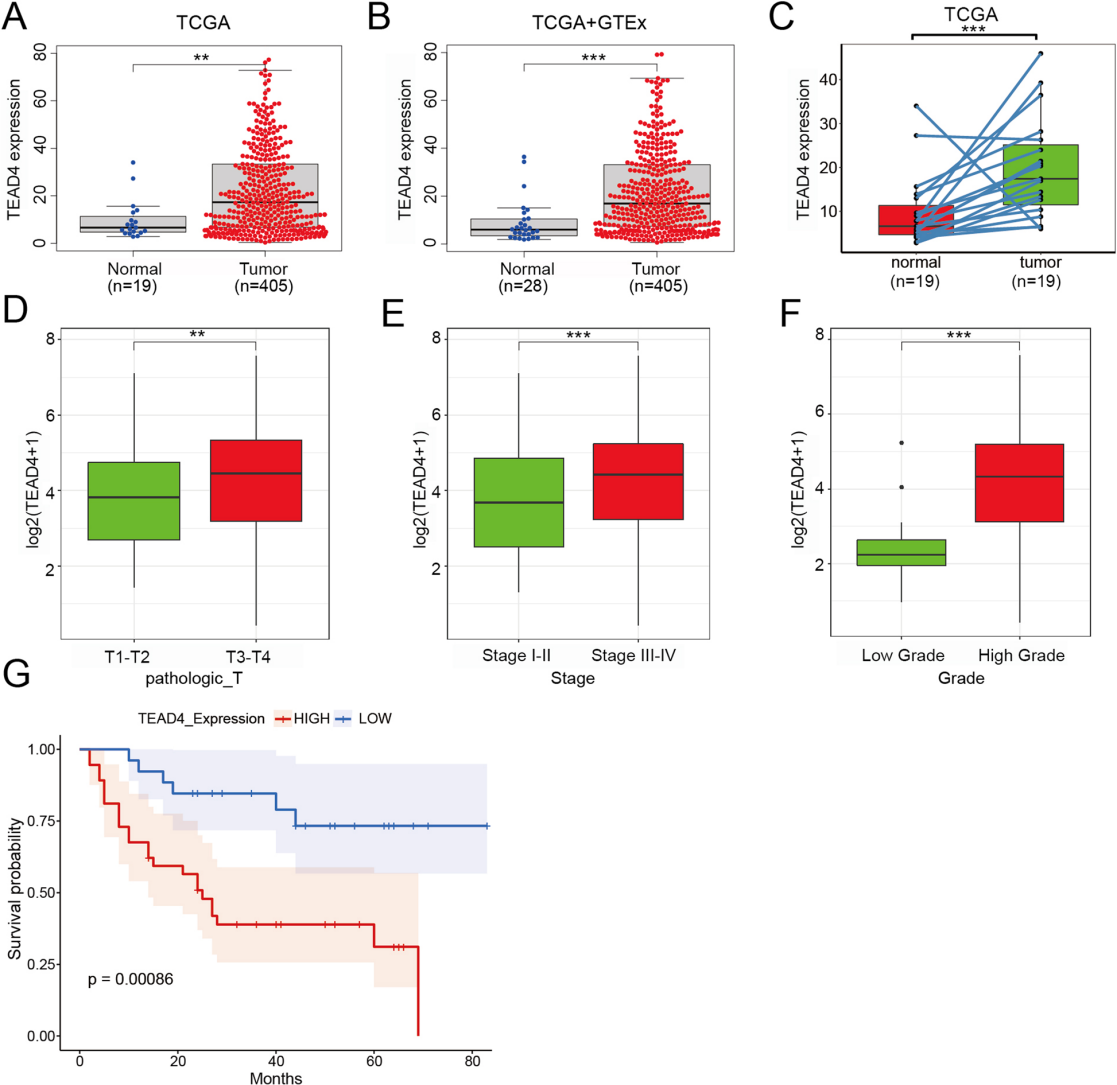
Analysis of TEAD4 expression and its clinical correlation in BLCA tissues from TCGA database (**A**) Relative mRNA expression of TEAD4 in the TCGA-BLCA database. (**B**) Relative mRNA expression of TEAD4 in TCGA-BLCA + GTEx database. (**C**) Relative mRNA expression of TEAD4 in BLCA tissues and their paired normal tissues from the TCGA database. (**D**) TEAD4 mRNA expression level in different pT stage of BLCA samples from TCGA. (**E**) TEAD4 mRNA expression level in different tumor stage of BLCA samples from TCGA. (**F**) TEAD4 mRNA expression level in different tumor grade of BLCA samples from TCGA. Data are expressed as mean ± SD (*p < 0.05; **p < 0.01; ***p < 0.001). (**G**) Kaplan-Meier survival curves of BLCA patients with high and low expression of TEAD4

To further explore the unique prognostic and potential therapeutic value of TEAD4 in BLCA, we first surveyed the expression of TEAD4 between the tumor tissues and the adjacent normal tissues using TCGA-BLCA and GTEx datasets. As shown in Fig. 4A and B, the expression of TEAD4 in BLCA was significantly higher than that in the adjacent tissues. In addition, TEAD4 was also obviously upregulated in the BLCA tissues compared to their paired normal tissues (Fig. 4C). Meanwhile, we estimated the association between the expression of TEAD4 and clinical characteristics. Compared to the T1-T2 stage, Stage I-II and low-grade groups, TEAD4 was highly expressed in the T3-T4 stage (*P* < 0.01), Stage III-IV (*P* < 0.001), and high-grade groups (*P* < 0.0001) (Fig. 4D-F). To further prove these findings, 163 patients with complete clinical information in the TCGA dataset were collected and analyzed to determine the levels of TEAD4 from different clinical stages. The consistent results were concluded in Supplementary Table 4.

To assess the role of TEAD4 in BLCA, we interrogated a BLCA TMA to determine its expression in this cancer and the association of its expression with patient survival, tumor grade (Low grade, High grade), tumor stage (stage I-IV), T stage (carcinoma in situ [Tis], Ta, T1–T4) and N stage (N0, N1). IHC staining of TEAD4 expression in these tissues were conducted and scored to explore the expression pattern. Kaplan Meier (K-M) survival analysis showed that the patients with higher TEAD4 levels tended to have worse OS (Fig. 4G). Further analyses revealed that TEAD4 expression was remarkably correlated with N stage and tumor grade (Table 1). Moreover, Cox analyses were conducted to explore the independent prognostic factors for OS (Table 2). Based on the univariate analysis, T stage, N stage, tumor stage and TEAD4 expression were identified as independent prognostic factors for OS; while only TEAD4 were evaluated and identified as independent prognostic factors for OS by both univariate and multivariate analysis. Taken together, these results showed that TEAD4 may be an effective biomarker for poor prognosis in BLCA.

**Table 1:** The association between TEAD4 levels and clinicopathologic features of BLCA patients (*n* = 63)

Parameters	Category	TEAD4 expression		*P* value
		High(*n* = 37)	Low(*n* = 26)	(Pearson's χ^2^)
Age	> 60	27	21	0.592
	< 60	9	5	
	NA	1	0	
Gender	Female	6	4	0.929
	male	31	22	
Grade	Low grade	3	4	0.433
	High grade	31	22	
	NA	3	0	
T	T1-T2	15	15	0.096
	T3-T4	20	8	
	NA	2	3	
N	N0	22	23	**0.043**
	N1	7	1	
	NA	8	2	
Stage	I-II	9	13	**0.023**
	III-IV	23	9	
	NA	5	4	

Abbreviations: *NA* Not available

Numbers in bold indicate *p* value with statistical significance

**Table 2:** Univariate and multivariate analysis of clinicopathological factors associated with OS (*n* = 63)

Clinicopathological	Univariate Analysis		Multivariate Analysis	
	HR (95% CI)	*P* value	HR (95% CI)	*P* value
Age	1.018	0.398	1.005	0.835
(< 60 vs ≥ 60)	(0.977–1.060)		(0.963–1.048)	
Gender	0.623	0.308	0.377	0.119
(Female vs Male)	(0.251–1.546)		(0.111–1.283)	
Grade	1.756	0.445	0.189	0.069
(Low vs High)	(0.414–7.440)		(0.031–1.142)	
T	2.174	**0.041**	2.066	0.431
(T1-T2 vs T3-T4)	(1.032–4.578)		(0.340–12.558)	
*N*	2.889	**0.042**	2.199	0.259
(N0 vs N1)	(1.041–8.018)		(0.559–8.639)	
Stage	3.647	**0.007**	1.927	0.549
(I-II vs III-IV)	(1.435–9.269)		(0.226–16.442)	
TEAD4 Expression	4.112	**0.002**	3.786	**0.015**
(Low vs High)	(1.673–10.106)		(1.292–11.091)	

Abbreviations: *HR* Hazard ratio, *CI* Confidence interval

Numbers in bold indicate *p* value with statistical significance

### TEAD4 promotes metastasis and invasion in BLCA cells

**Fig. 5:**
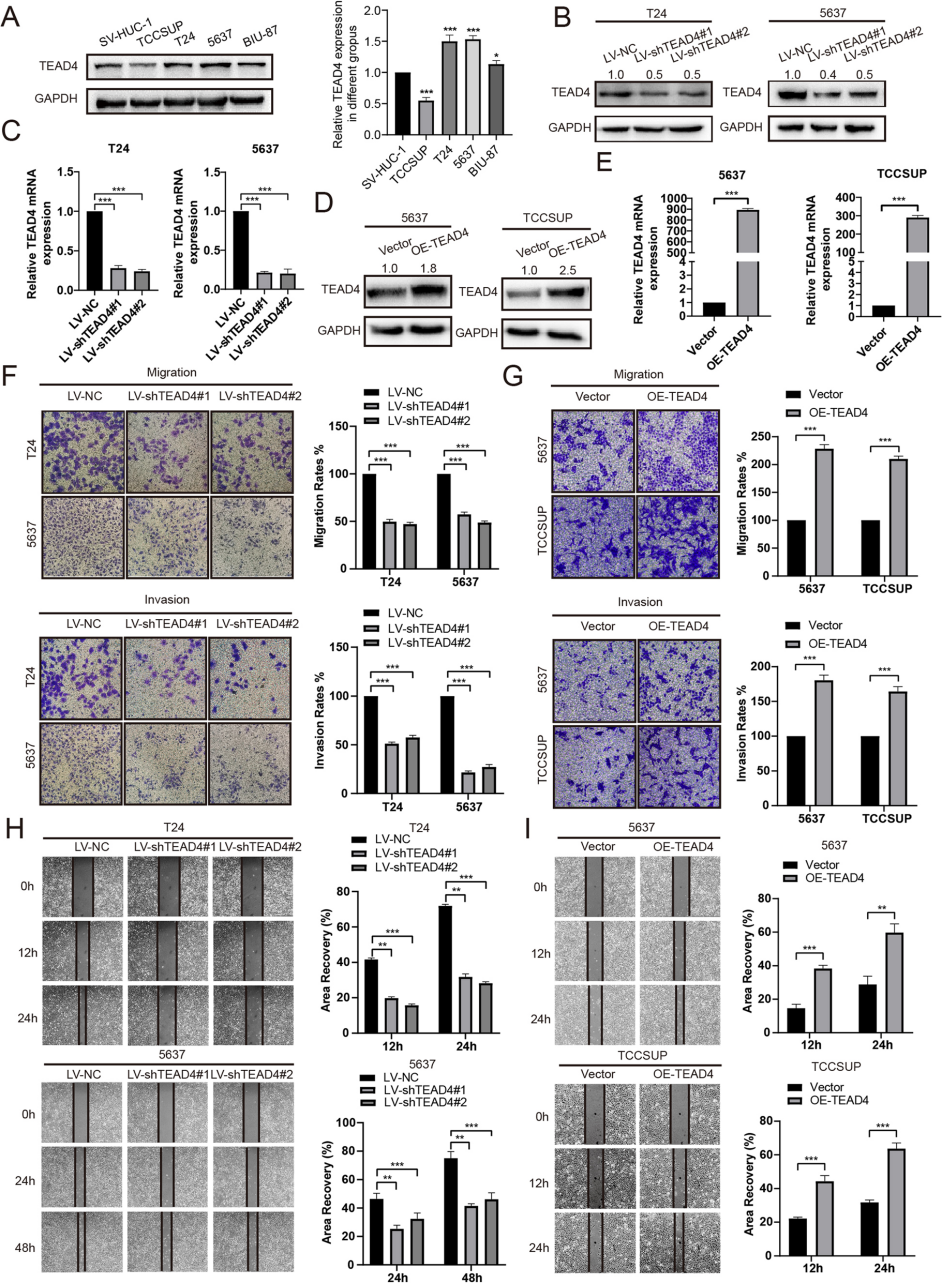
TEAD4 enhances the metastatic and invasion potential of BLCA cells (**A**) Relative protein expression of TEAD4 in normal bladder cell SV-HUC1 and four BLCA cell lines, as detected by immunoblot analysis. (**B**, **C**) RT-qPCR and western blot analysis of TEAD4 expression level in the BLCA cells transfected with LV-NC or LV-shTEAD4. (**D**, **E**) rt-PCR and western blot analysis of TEAD4 expression level in the BLCA cells transfected with Vector or OE-TEAD4 plasmid. (**F**) Representative data from Transwell migration and Matrigel invasion assays performed with the TEAD4 knock down cells (T24, 2 × 10^4^ cells, 24 h; 5637, 4 × 10^4^ cells, 36 h). (**G**) Representative data from Transwell migration and Matrigel invasion assays performed with the TEAD4 overexpression cells (5637, 4 × 10^4^ cells, 24 h; TCCSUP, 4 × 10^4^ cells, 36 h). (**H**) Representative data from wound healing migration assays performed with the TEAD4 knock down cells. (**I**) Representative data from wound healing migration assays performed with the TEAD4 overexpression cells. Data are expressed as mean ± SD (*p < 0.05; **p < 0.01; ***p < 0.001)

The basic protein expression of TEAD4 in BLCA cell lines (SV-HUC-1, 5637, BIU-87, T24, and TCCSUP) was detected with Western Blot method. The results showed that relative TEAD4 protein levels were higher in 5637, Biu-87 and T24 cells compared with that in normal bladder cell line SV-HUC1 (Fig. 5A). To further investigate the biological role of TEAD4 in BLCA cells, we knocked down the expression of TEAD4 in T24 and 5637 cells via LV-shTEAD4 or upregulated the TEAD4 levels in TCCSUP and 5637 cells, respectively, depending on the experimental purpose (Fig. 5B-E). The proliferation ability of BLCA cells was significantly affected neither by TEAD4 overexpression nor by TEAD4 knockdown, which was confirmed by both clone formation and cck-8 assays (Supplementary Fig. 4).

Wound healing and trans-well assays were used to detect changes in the invasion and migration ability of BLCA cells. The results proved that TEAD4 knockdown significantly impaired the invasion and migration ability in T24 and 5637 cells, while TEAD4 overexpression had the opposite effects in TCCSUP and 5637 cells (Fig. 5F-I). To further confirm the biological function of TEAD4 in BLCA, we restroed TEAD4 expression in the cells that TEAD4 was stably knocked down (Supplementary Fig. 5A). Next, migration and invasion assays were conducted and showed that the restored expression of TEAD4 rescued the migratory and invasive abilities of TEAD4-knockdown BLCA cells (Supplementary Fig. 5B and C). Collectively, these results indicated that TEAD4 could promote the invasion and metastasis of BLCA cells.

### TEAD4 activates EMT in BLCA cells

**Fig. 6:**
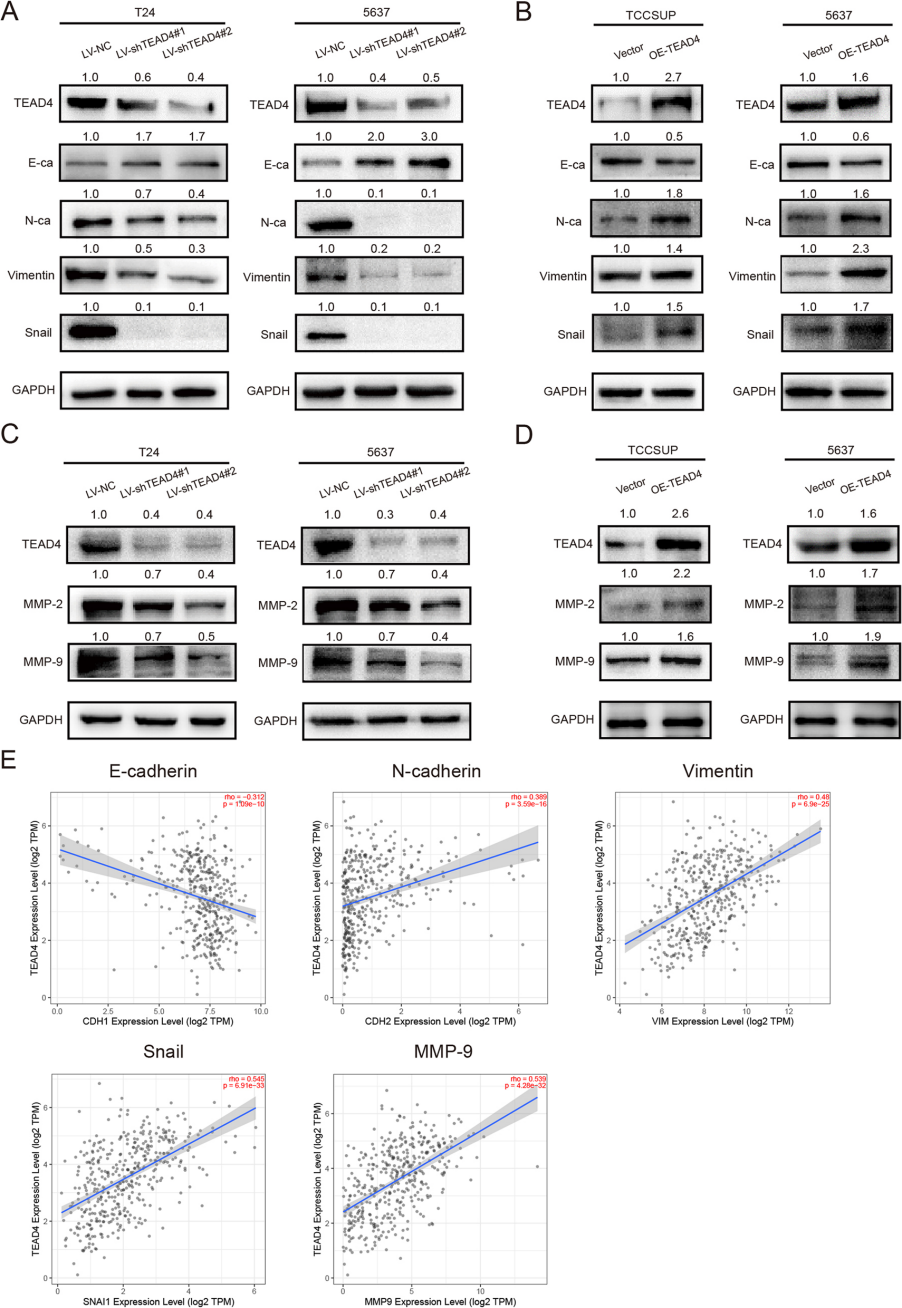
TEAD4 activates EMT in BLCA cells (**A**) Relative expression levels of E-cadherin, N-cadherin, Vimentin, Snail, TEAD4 and GAPDH in the T24 and 5637 cells transfected with LV-NC or LV-shTEAD4. (**B**) Relative expression levels of E-cadherin, N-cadherin, Vimentin, Snail, TEAD4 and GAPDH in the TCCSUP and 5637 cells transfected with Vector or OE-TEAD4 plasmid. (**C**) Relative expression levels of TEAD4, MMP-2, MMP-9 and GAPDH in the T24 and 5637 cells transfected with LV-NC or LV-shTEAD4. (**D**) Relative expression levels of TEAD4, MMP-2, MMP-9 and GAPDH in the TCCSUP and 5637 cells transfected with Vector or OE-TEAD4 plasmid. (**E**) Correlation analysis between TEAD4 and E-cadherin, N-cadherin, Vimentin, Snail or MMP-9 mRNA expression level by TIMER2.0 online database (*n* = 408)

Our analysis results above suggested that TEAD4 was positively correlated with EMT, so the expression changes of EMT markers were detected when TEAD4 levels were altered in the BLCA cells. The protein level of the epithelial marker E-cadherin was notably increased while the expression of mesenchymal markers, such as N-cadherin, Vimentin Snail and MMP-2/9, were considerably decreased in TEAD4 knockdown cells, which were totally inverted in the TEAD4 overexpressed cells (Fig. 6A -D). In addition, we used the TIMER2.0 online database to analyze the correlation between TEAD4 and EMT-related genes and found that TEAD4 was also negatively correlated with E-cadherin and positively correlated with N-cadherin, vimentin, snail and MMP-9 at mRNA levels (Fig. 6E).

These results indicated that TEAD4 triggered EMT in BLCA cells.

### TEAD4 boosts PI3K/AKT pathway in BLCA cells

**Fig. 7:**
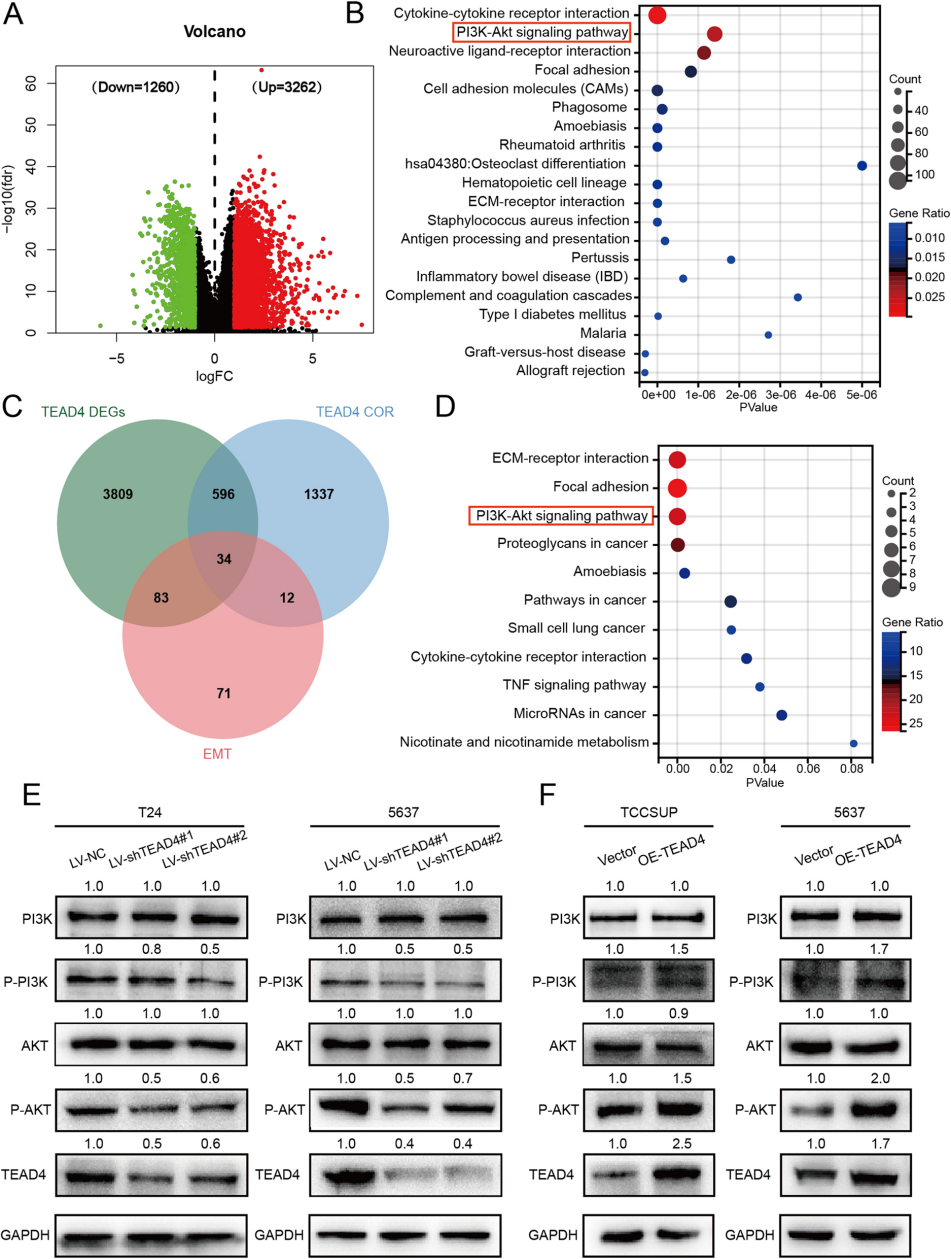
TEAD4 regulates the PI3K/AKT pathway in BLCA cells (**A**) Volcano plot of DEGs extracted by TEAD4 mRNA expression. (*P* < 0.05, |log2 FC|≥ 1). (**B**) KEGG analysis for 4522 DEGs extracted by TEAD4 mRNA expression. (**C**) Venn diagram of the EMT genes that are strongly correlated with TEAD4, revealing 34 intersected genes. (**D**) KEGG analysis for 34 genes that coexisted in the three gene sets. (**E**) Relative expression levels of PI3K, AKT, p-PI3K, p-AKT, TEAD4 and GAPDH in the T24 and 5637 cells transfected with LV-NC or LV-shTEAD4. (**F**) Relative expression levels of PI3K, AKT, p-PI3K, p-AKT, TEAD4 and GAPDH in the 5637 and TCCSUP cells transfected with Vector or OE-TEAD4 plasmid

In order to clarify the mechanism by which TEAD4 regulates the EMT of BLCA cells, TCGA-BLCA samples were divided into high—expression and low—expression groups according to the median TEAD4 level. A total of 4522 DEGs, 3262 up-regulated and 1,260 down-regulated, were identified (Fig. 7A). DEGs were radically enriched in 20 pathways through KEGG analysis, among which PI3K/AKT signaling pathway was most closely related to EMT (Fig. 7B).

To obtain the EMT genes that are strongly correlated with TEAD4 in BLCA, 1979 genes that dramatically correlated with TEAD4 were extracted using limma R package based on Spearman algorithm. The absolute value of correlation coefficient was set to > 0.3. Meantime, EMT hallmark gene set including 200 genes was obtained from the Molecular Signatures Database (version 7.2). Finally, 34 genes that coexisted in the three gene sets were selected for further analysis (Fig. 7C). KEGG enrichment analysis on these 34 genes showed that PI3K/AKT signaling pathway was remarkably enriched, too (Fig. 7D).

To confirm the promotive effects of PI3K/AKT pathway on EMT in BLCA cells, T24 and 5637 cells were treated with LY294002 (20uM), a PI3K/AKT inhibitor, for 24 h. The protein level of E-cadherin was increased, and the protein levels of N-cadherin, Vimentin and Snail were decreased (Supplementary Fig. 6A) in the cells treated with LY294002. Wound healing and Trans-well assays showed that the migration and invasion ability of T24 and 5637 cells were markedly attenuated by LY294002 (Supplementary Fig. 6B and C). The mentioned results indicated that the PI3K/AKT pathway is involved in regulating the EMT of BLCA cells.

Via western blot, We observed that the protein levels of phospho-PI3K and phospho-AKT in the TEAD4 knockdown cells were remarkedly inhibited, which were elevated in the TEAD4 overexpressed cells, with total PI3K and AKT expressions unchanged (Fig. 7E-F).

### TEAD4 regulates EMT of BLCA cells via PI3K/AKT pathway

**Fig. 8:**
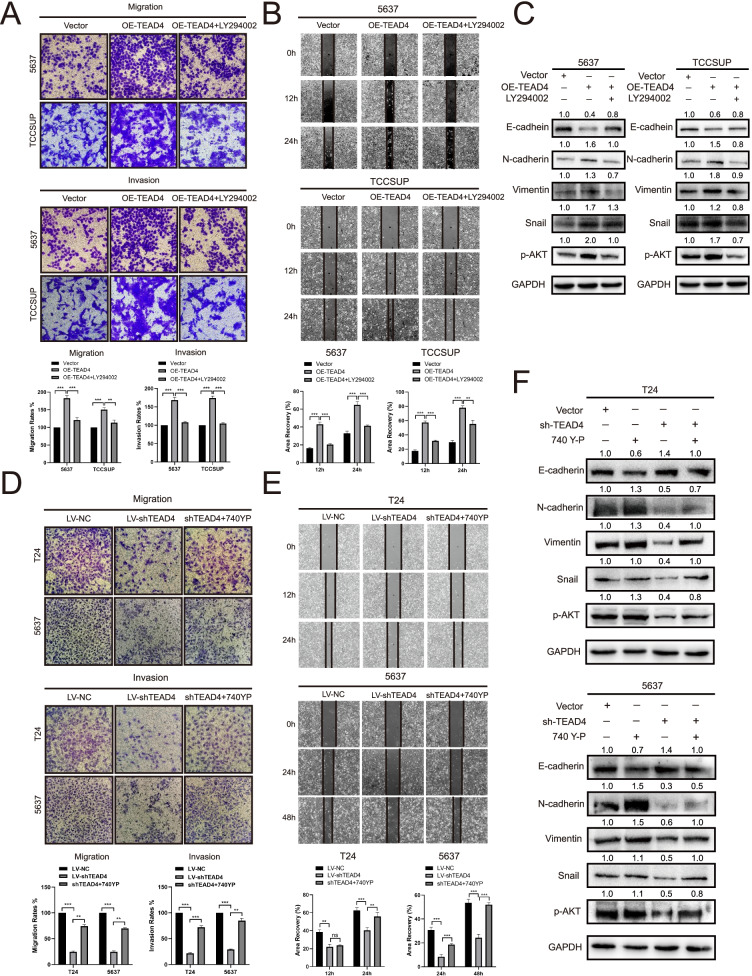
TEAD4 promotes EMT in BLCA cells via PI3K/AKT signaling pathway (**A**) Representative data from Transwell migration and Matrigel invasion assays performed in the indicated cells treated with or without LY294002 (5637, 4 × 10^4^ cells, 24 h; TCCSUP, 4 × 10^4^ cells, 48 h). (**B**) Representative data from wound healing migration assays performed in the indicated cells treated with or without LY294002. (**C**) Relative expression levels of E-cadherin, N-cadherin, Vimentin, Snail, p-AKT and GAPDH in the indicated cells treated with or without LY294002. (**D**) Representative data from Transwell migration and Matrigel invasion assays performed in the indicated cells treated with or without 740Y-P (T24, 2 × 10^4^ cells, 36 h; 5637, 4 × 10^4^ cells, 36 h). (**E**) Representative data from wound healing migration assays performed in the indicated cells treated with or without 740Y-P. (**F**) Relative expression levels of E-cadherin, N-cadherin, Vimentin, Snail, p-AKT and GAPDH in the indicated cells treated with or without 740Y-P. Data are expressed as mean ± SD (*p < 0.05; **p < 0.01; ***p < 0.001)

To determine whether the activation of PI3K/AKT pathway is essential for the TEAD4-mediated promotion of EMT, we treated TEAD4-overexpressed BLCA cells with LY294002. We found that the TEAD4-mediated enhancement of cell invasion and migration was abolished by LY294002 (Fig. 8A and B). The expression levels of E-cadherin, N-cadherin, Vimentin, and snail in LY294002-treated cells was significantly constrained compared with those in TEAD4-overexpressed cells (Fig. 8C).

Then, TEAD4-knockdown BLCA cells were treated with a PI3K/AKT pathway activator (740Y-P, 20uM). We took note of the fact that the abolished ability of invasion and migration in TEAD4-knockdown cells was obviously restored with the addition of 740Y-P (Fig. 8D and E). Western blot indicated that the expression of E-cadherin, N-cadherin, Vimentin, and snail was rescued by the addition of 740Y-P in TEAD4-knockdown cells (Fig. 8F).

Based on these findings, we speculated that TEAD4 could regulate EMT by activating the PI3K/AKT pathway in BLCA cells.

### TEAD4 enhances metastasis of BLCA cells in vivo

**Fig. 9:**
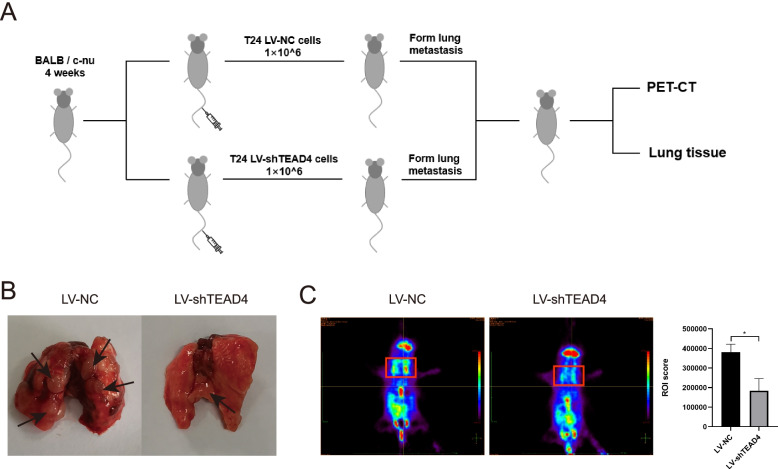
TEAD4 knockdown suppressed the BLCA cells distant metastasis in vivo (**A**) Schematic diagram of a metastatic model of nude mice with T24 cells injected into the tail vein. (**B**) The general appearance of the lungs from the LV-shTEAD4 and LV-NC groups. (**C**) Representative PET-CT images from the LV-shTEAD4 and LV-NC groups are shown in the left panel, Statistics of ROI Score are shown in the right panel. Data are expressed as mean ± SD (*p < 0.05; **p < 0.01; ***p < 0.001)

To further validate the metastasis-promoting roles of TEAD4 in BLCA cells, we constructed a lung metastasis model by injecting T24 cells stable transfected with LV-NC or LV-shTEAD4 into the tail veins of nude mice (1 × 10^6^ cells per mouse, *n* = 6 for each group) (Fig. 9A). Eight weeks after injection, we observed that there were fewer microscopic metastatic nodules in the LV-shTEAD4 group than those in the LV-NC group (Fig. 9B). At the same time, the pulmonary metastasis was confirmed by PET-CT. PET-CT using [18F]-FDG revealed significantly decreased uptake values in the LV-shTEAD4 group than that in the LV-NC group (Fig. 9C), demonstrating that TEAD4 knockdown significantly reduced BLCA cell migration in vivo.

These mentioned results indicated that TEAD4 could promote the metastasis of BLCA cells in vivo.

## Discussion

In recent years, many studies have shown that EMT is one of the key processes in promoting tumor metastasis [7, 31]. EMT is also a key event in its metastasis in BLCA, which has brought us a great clinical challenge for the treatment of this maligancy. Therefore, it is becoming increasingly acute and important to find the molecule that has to be not only a prognosis predictor but also a target for the regulation of metastasis of BLCA.

With the development of high-throughput sequencing in recent decades, our understanding of BLCA biology has been largely improved [32–34]. An increasing number of molecules involved in BLCA progression have been screened through bioinformatics analysis [35]. In this study, we aimed to find a powerful predictor of metastasis and poor prognosis of BLCA. Firstly, overlapping DEGs were screened out by analyzing the expression profiles from GTEx and TCGA BLCA datasets, among which four independent prognostic factors (FASN, IGFL2, PLOD1 and TEAD4) were finally determined by Kaplan–Meier survival and Cox regression analyses. Synchronously, three valuable clinical factors closely related to prognosis of BLCA were determined by multivariate Cox regression analysis, subject to which a prognostic risk prediction model for BLCA was constructed. According to the median risk score, TCGA BLCA samples were divided into notbaly different low- and high-risk groups; GSEA analysis showed that EMT pathway was the top enriched gene signature in the high-risk group, which proved that EMT was a key factor leading to poor prognosis of BLCA.

A recent bioinformatics study found that EMT played a key role in the transition from NMIBC to MIBC and was closely associated with poor prognosis of BLCA [12]. Previous experimental studies showed that EMT was enhanced during the malignant transition from NMINC to MIBC [36]. These results proved that EMT also had a crucial role in BLCA progression and metastasis. Here, we selected the EMT-related genes for the NMF cluster analysis, base on which we divided the TCGA-BLCA samples into two clusters according to the degree of EMT correlation. Kaplan Meier Analysis was used to compare overall survival rates between the two clusters. It has been revealed that high degree of EMT was associated with poor prognosis of BLCA patients.

To clarify the relationship between the expressions of FASN, IGFL2, PLOD1 and TEAD4 and EMT, ssGSEA analysis using EMT-derived signatures was conducted. The EMT signature-derived ssGSEA scores showed that TEAD4 had the strongest positive correlation with EMT in BLCA. Similar results were observed in some other studies, which found that TEAD4 could promote EMT in cancer cells, such as glioma, head neck squamous cell carcinoma, colorectal cancer, etc [16, 37–39]. In our previous study, we found that metformin could inhibit the proliferation of BLCA cells by regulating the YAP1/TEAD4 complex [40]. Now, we systematically analyzed the potential biological function of TEAD4 in bladder cancer and found that TEAD4 was highly expressed in BLCA and negatively correlated with OS in BLCA patients. Analysis on the clinical data from TCGA-BLCA datasets showed that TEAD4 expression was closely associated with tumor stage and grade. Then, we constructed TEAD4 stable knockdown cells lines with lentiviral vector-based short hairpin RNA (shRNA) and TEAD4 overexpressed cell lines with TEAD4 ectopic expression plasmids for in vitro validation. Interestingly, we found that TEAD4 expression significantly strengthened the ability of invasion and metastasis of BLCA cells. It also has been confirmed that TEAD4 knockdown dramatically inhibited the lung tumor metastasis of BLCA cells in the in-vivo models of metastasis. Simultaneously, our results showed that the expression of N-cadherin, vimentin, and snail was down-regulated when TEAD4 was silenced. Consistent with our results, it was proved that TEAD4 knockdown inhibited the expressions of mesenchymal markers including N-CA, FN1 and TWIST1/2 in BLCA cells [18]. All results provided evidence that TEAD4 was a vital regulator of EMT in BLCA cells.

Numerous studies have found that the activated PI3K/AKT signaling pathway is closely related to the invasion and metastasis of tumor cells [20]. In BLCA, GAL1, ZNF139 and circZNF139 promoted the invasion and metastasis all by the activation of PI3K/AKT pathway [41, 42]. Our KEGG enrichment analysis of genes related to TEAD4 expression and EMT—related genes showed that the enrichment was significantly concerned with PI3K/AKT pathway, suggesting that PI3K/AKT pathway was involved in TEAD4 regulated EMT process in BLCA. Following, we verified that TEAD4 expressoin could increase the phosphorylation level of PI3K and AKT, suggesting TEAD4 activated PI3K/AKT pathway in BLCA cells.

In contrast to the past studies that have demonstrated that EMT was a key biological process leading the subtype transition from NMIBC to MIBC, we also proved that EMT is a vital factor that leads to poor outcomes of BLCA patients based on our prognostic risk model established by the clinical characters, which has been validated reversely via our clustering analysis. Through DEGs screening and survival analysis followed by EMT correlation analysis, TEAD4 has been systematically identified as the pivotal biomarker that strongly associated with EMT and poor prognosis. Although a recent study proved experimentally that TEAD4 was related to EMT in BLCA cells, the concrete mechanism by which TEAD4 was involved in EMT has not been clarified. Here, we first elucidated that TEAD4-mediated EMT activation is by means of stimulating PI3K/AKT pathway in BLCA cells. Concurrently, we confirmed that TEAD4 expression augments BLCA cell metastasis both in vitro and in vivo. Nevertheless, the manner in which TEAD4 regulates PI3K/AKT pathway and the specific and direct target(s) of TEAD4 that controls PI3K/AKT pathway are not interpreted here and needs to be studied further.

In conclusion, we found that TEAD4 is a powerful predictor of poor prognosis and promotes EMT of BLCA cells through activating PI3K/AKT pathway, resulting in the enhancement of cell migration and invasion. These findings provide not only an effective biomarker for predicting the prognosis but also a great potential target for treatment of metastatic BLCA.

## Supplementary Information

**Additional file 1. **Supplementary Figures 

**Additional file 2. **Supplementary Table  

## Data Availability

The datasets used and/or analyzed during the current study are available from the corresponding author on reasonable request.

## Abbreviations

BLCA: Bladder cancer
EMT: Epithelial—mesenchymal transition
GSEA: Gene Set Enrichment Analysis
DEG: Differentially expressed genes
TEAD4: TEA domain transcription factor 4
FASN: Fatty acid synthase
IGFL2: IGF Like Family Member 2
PLOD1: Procollagen-Lysine, 2-Oxoglutarate 5-Dioxygenase 1
NMIBC: Non-muscle invasion bladder cancer
MIBC: Muscle invasive bladder cancer
GEO: Gene Expression Omnibus
TCGA: The Cancer Genome Atlas
GTEx: Genotype-Tissue Expression
KEGG: Kyoto Encyclopedia of Genes and Genomes
