# Supplementary material for: TEAD4 functions as a prognostic biomarker and triggers EMT via PI3K/AKT pathway in bladder cancer
Source: J Exp Clin Cancer Res. 2022 May 17;41:175. doi: 10.1186/s13046-022-02377-3 (PMC9112458; doi:10.1186/s13046-022-02377-3)
Supplement: Supplementary file 1 — Additional file 1. Supplementary Figures [file 13046_2022_2377_MOESM1_ESM.pptx]

## Slide 1
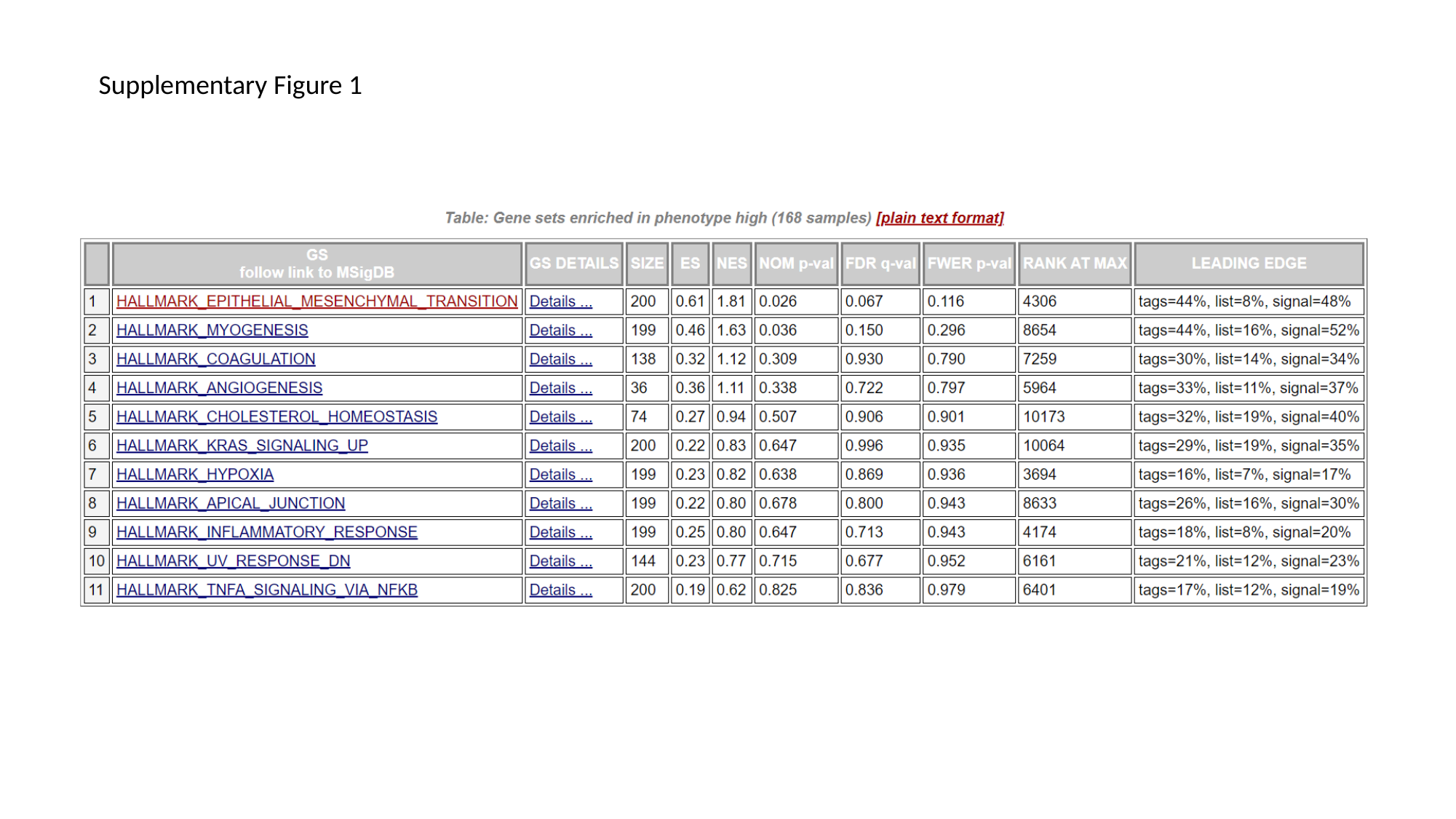

Supplementary Figure 1

## Slide 2
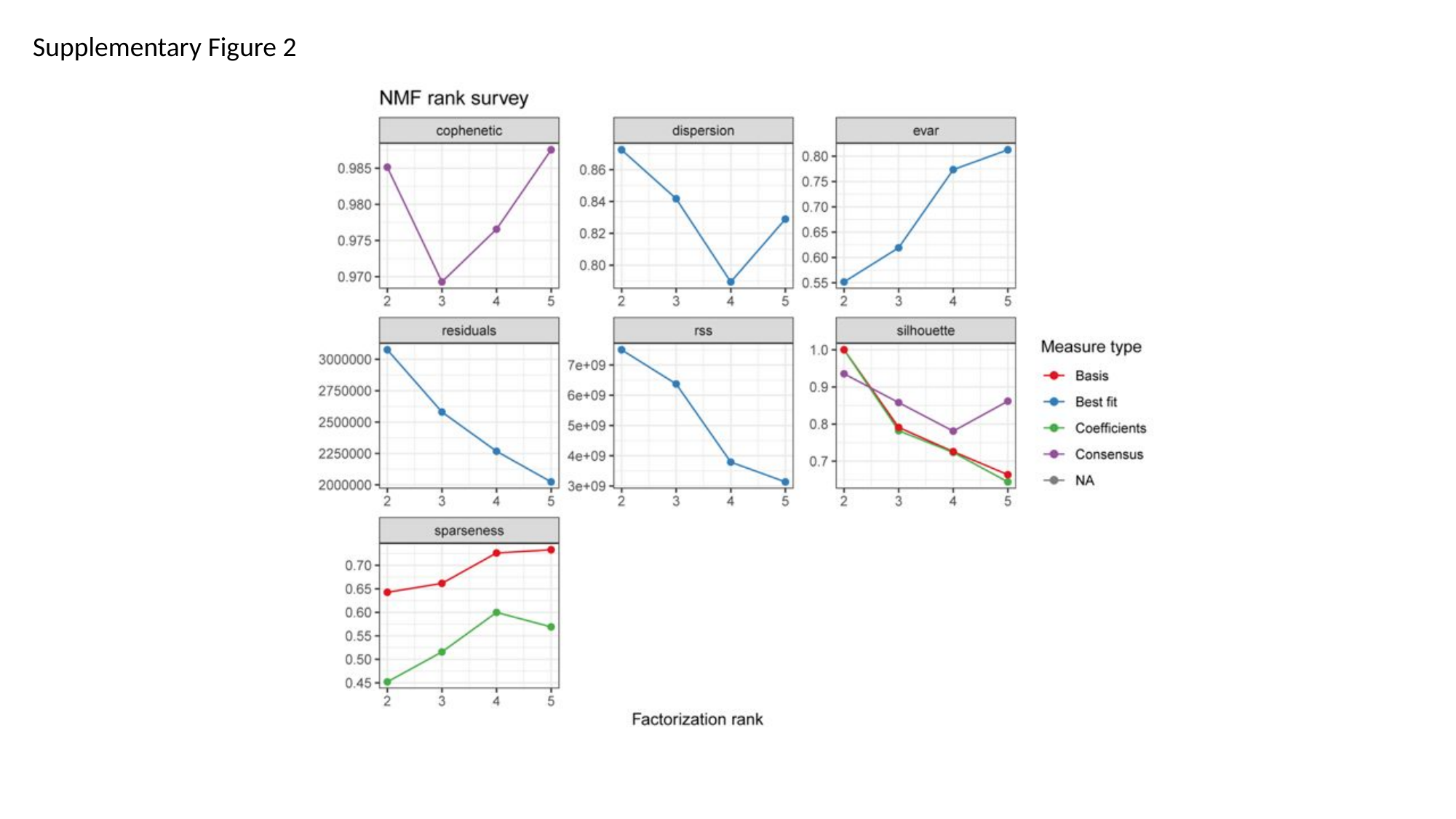

Supplementary Figure 2

## Slide 3
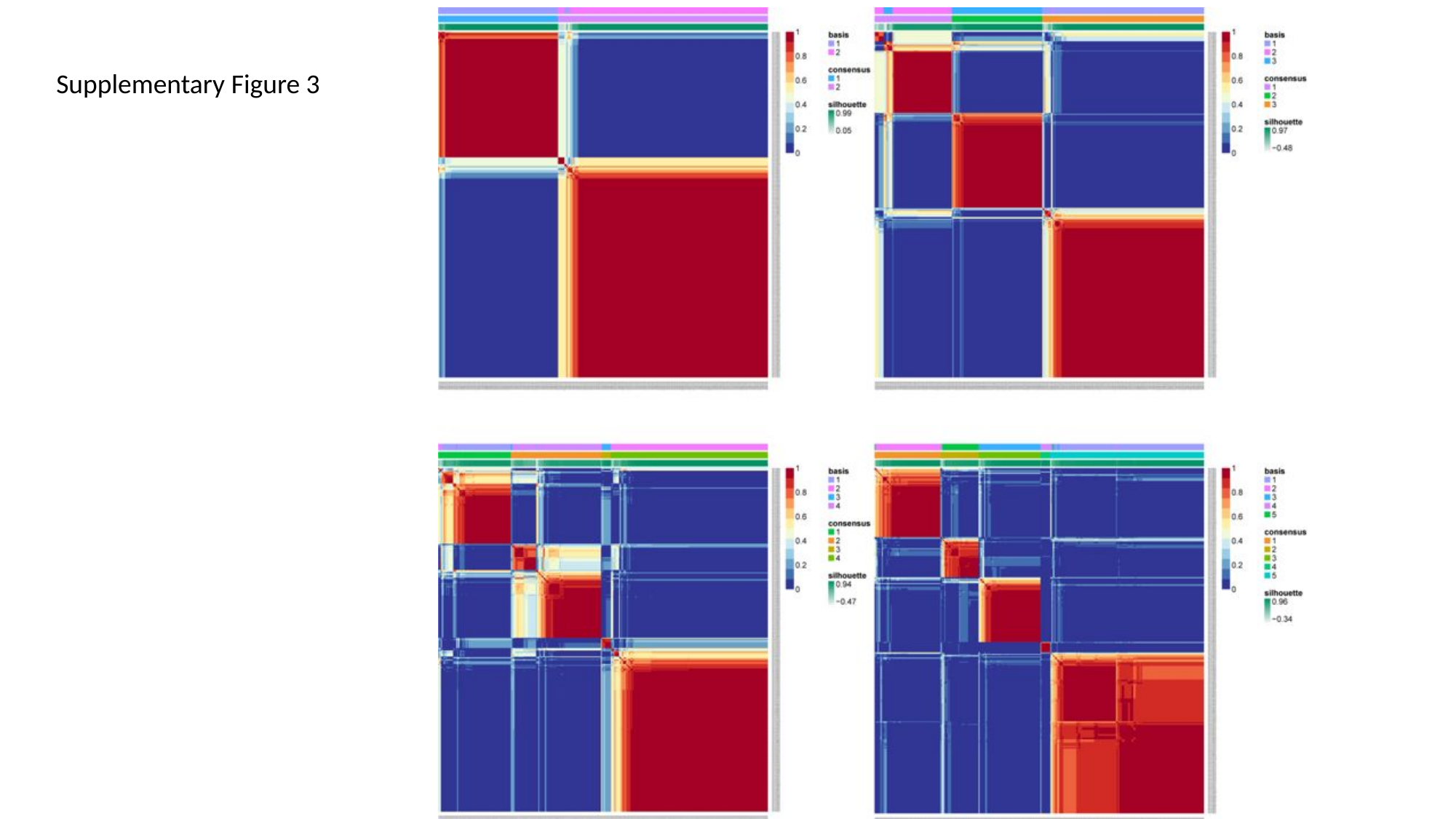

Supplementary Figure 3

## Slide 4
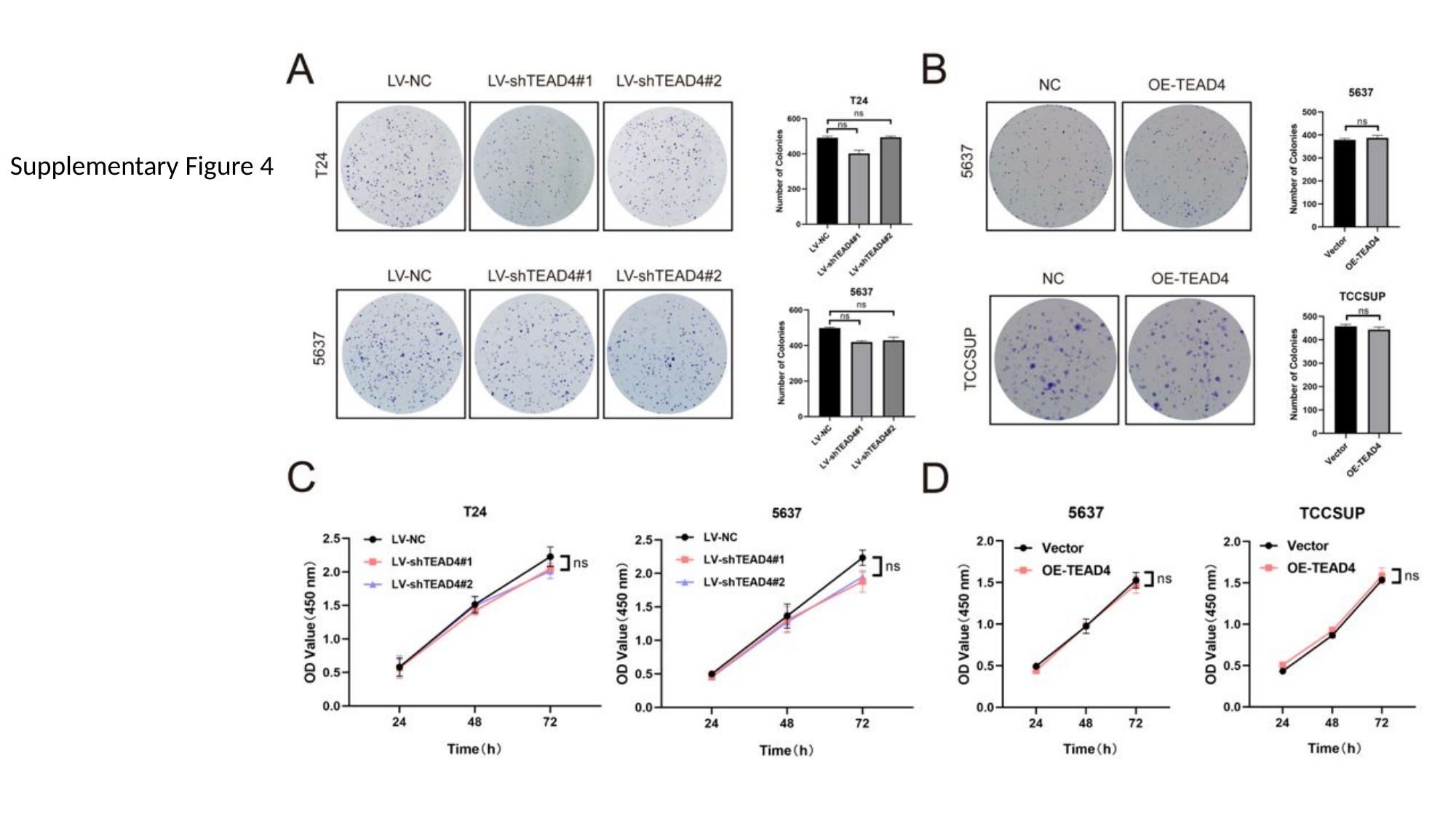

Supplementary Figure 4

## Slide 5
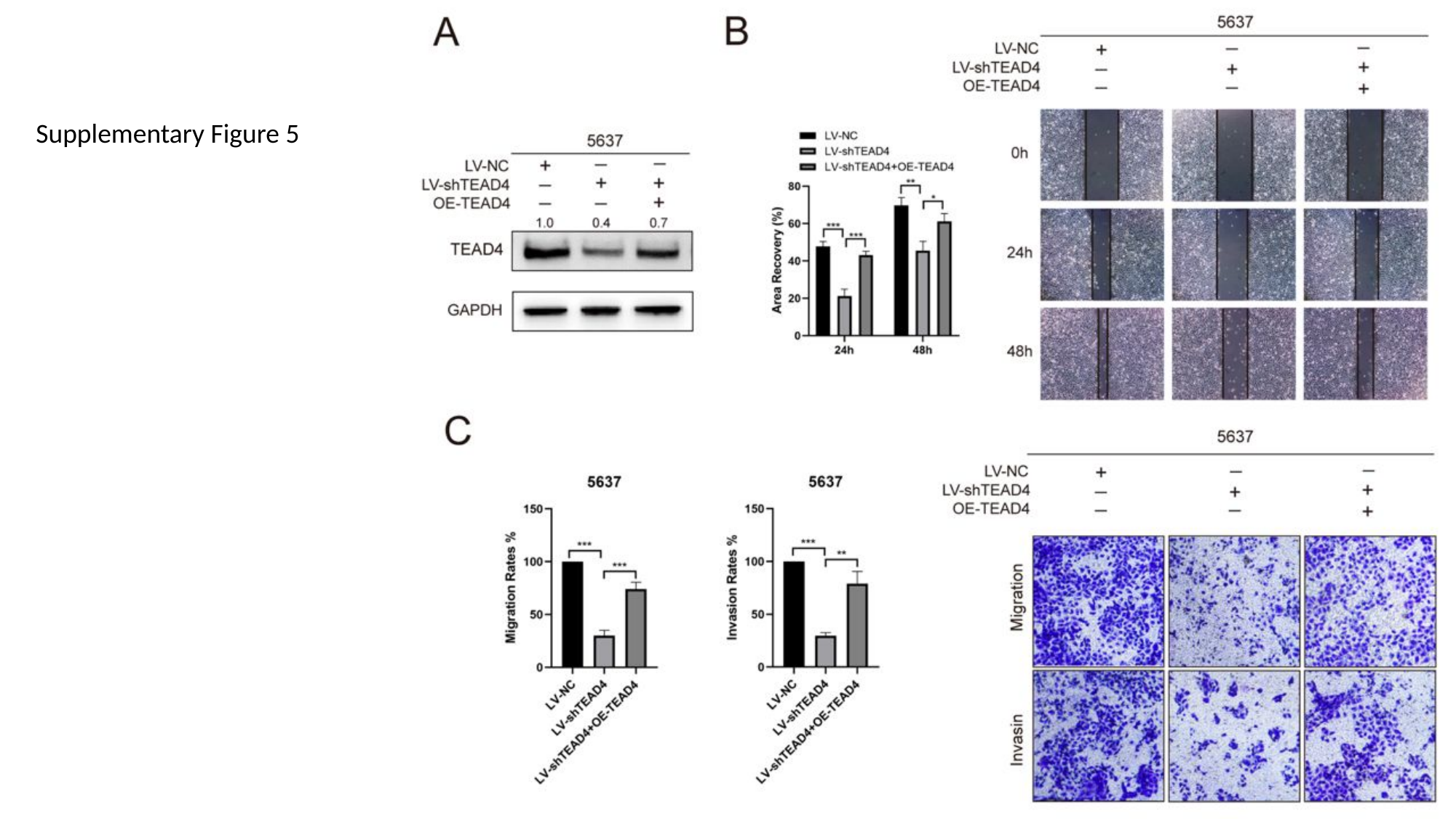

Supplementary Figure 5

## Slide 6
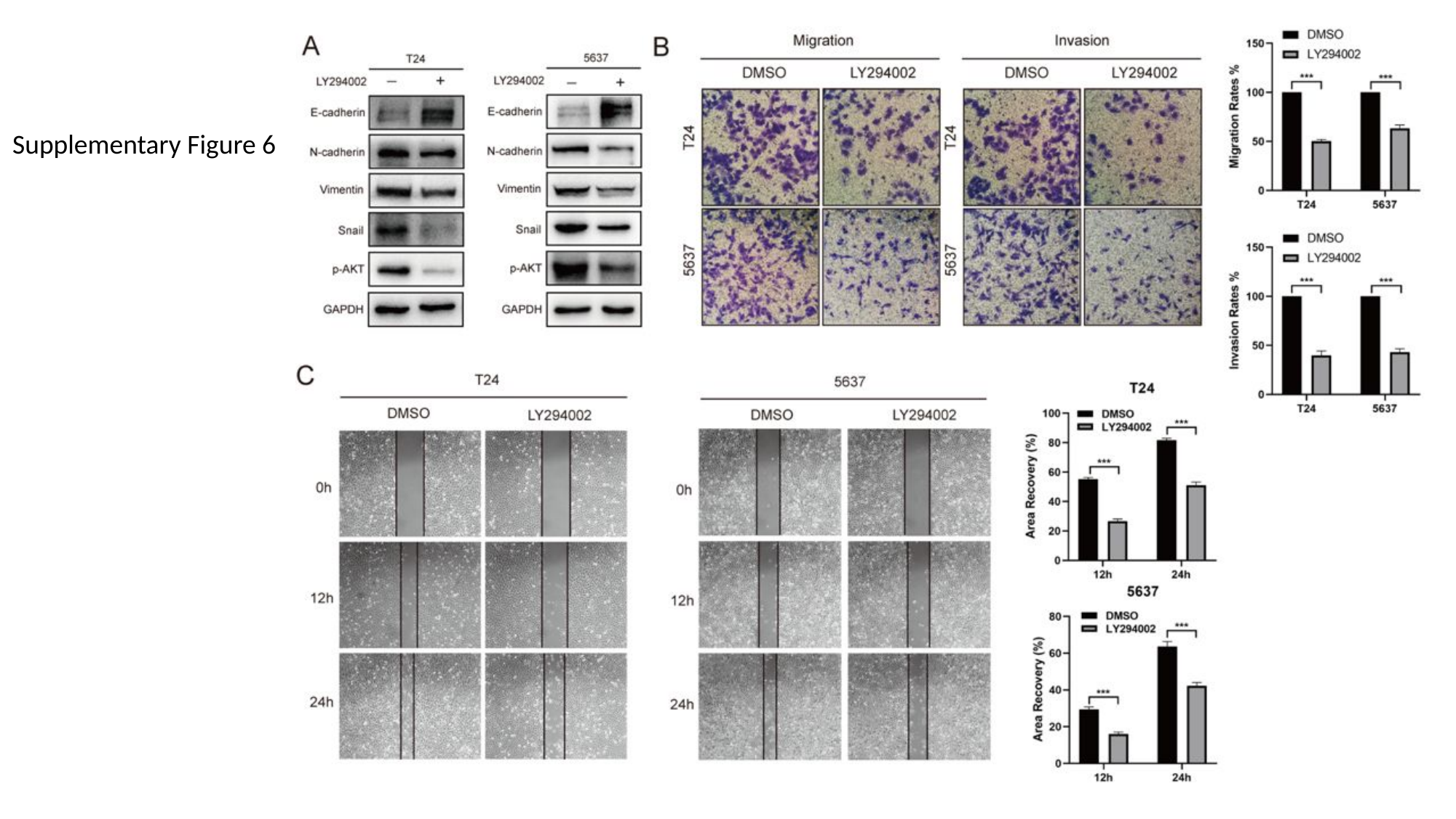

Supplementary Figure 6
