# Supplementary material for: TEAD4 functions as a prognostic biomarker and triggers EMT via PI3K/AKT pathway in bladder cancer
Source: J Exp Clin Cancer Res. 2022 May 17;41:175. doi: 10.1186/s13046-022-02377-3 (PMC9112458; doi:10.1186/s13046-022-02377-3)
Supplement: Supplementary file 2 — Additional file 2. Supplementary Table [file 13046_2022_2377_MOESM2_ESM.docx]

**Supplementary Table 1.**

**shRNA sequence：**

| ID | 5’ | stem | loop | stem | 3’ |
| --- | --- | --- | --- | --- | --- |
| TEAD4-RNAi(102054-1)-a | Ccgg | gaGACAGAGTATGCTCGCTAT | CTCGAG | ATAGCGAGCATACTCTGTCTC | TTTTTg |
| TEAD4-RNAi(102054-1)-b | aattcaaaaa | gaGACAGAGTATGCTCGCTAT | CTCGAG | ATAGCGAGCATACTCTGTCTC |  |
| TEAD4-RNAi(102055-1)-a | Ccgg | ccTTTCTCTCAGCAAACCTAT | CTCGAG | ATAGGTTTGCTGAGAGAAAGG | TTTTTg |
| TEAD4-RNAi(102055-1)-b | aattcaaaaa | ccTTTCTCTCAGCAAACCTAT | CTCGAG | ATAGGTTTGCTGAGAGAAAGG |  |

**Supplementary Table 2.**

**Primer sequence:**

| Gene | Forward primer | Reverse primer |
| --- | --- | --- |
| 18s | GCAGAATCCACGCCAGTACAAGAT | TCTTCTTCAGTCGCTCCAGGTCTT |
| TEAD4 | GAATGGACACTACTCTTACCGCATCC | AGCACGCTGTTCATCATGTACTTCTC |

**Supplementary Table 3.**

Screening for independent prognostic factors in four genes, FASN, IGFL2, PLOD1 and TEAD4

| ID | HR | HR.95L | HR.95H | p-value |
| --- | --- | --- | --- | --- |
| FASN | 1.397381 | 1.091913 | 1.788306 | **0.007846** |
| IGFL2 | 1.264608 | 1.101864 | 1.45139 | **0.000838** |
| PLOD1 | 1.538159 | 1.138154 | 2.078746 | **0.005077** |
| TEAD4 | 1.323597 | 1.085765 | 1.613525 | **0.005534** |

**Numbers in bold indicate p value with statistical significance.**

**Supplementary Table 4.**

The association between TEAD4 protein levels and clinicopathological features of BLCA patients

| characters | | TEAD4 | | t/ꭕ² | P value |
| --- | --- | --- | --- | --- | --- |
|  |  | Low | High |  |  |
| Age | | 66.22±10.772 | 68.75±9.607 | -1.579 | 0.116 |
| Gender | female | 18 | 17 | 0.188 | 0.664 |
|  | male | 70 | 56 |  |  |
| T | T1-T2 | 38 | 15 | 9.257 | **0.002** |
|  | T3-T4 | 50 | 58 |  |  |
| N | N0 | 65 | 45 | 2.753 | 0.097 |
|  | ≥N1 | 23 | 28 |  |  |
| M | M0 | 85 | 69 | 0.411 | 0.521 |
|  | M1 | 3 | 4 |  |  |
| Stage | I-II | 35 | 13 | 9.199 | **0.002** |
|  | III-IV | 53 | 60 |  |  |
| Grade | Low grade | 14 | 1 | 9.983 | **0.002** |
|  | High grade | 74 | 72 |  |  |
| OS | Alive | 66 | 38 | 9.186 | **0.002** |
|  | Dead | 22 | 35 |  |  |
| OS.time | | 902.68±891.368 | 804.26±965.845 | 0.666 | 0.506 |
| in total | | 88 | 73 |  |  |

**Numbers in bold indicate p value with statistical significance.**
